# Effect of Chinese Herbal Medicines on Hearing Loss Risk in Rheumatoid Arthritis Patients: Retrospective Claims Analysis

**DOI:** 10.3389/fmed.2021.683211

**Published:** 2021-07-20

**Authors:** Hsin-Hua Li, Hanoch Livneh, Wei-Jen Chen, Wen-Lin Fan, Ming-Chi Lu, How-Ran Guo, Tzung-Yi Tsai

**Affiliations:** ^1^Department of Chinese Medicine, Dalin Tzuchi Hospital, The Buddhist Tzuchi Medical Foundation, Chiayi, Taiwan; ^2^Department of Environmental and Occupational Health, College of Medicine, National Cheng Kung University, Tainan, Taiwan; ^3^Rehabilitation Counseling Program, Portland State University, Portland, OR, United States; ^4^Graduate Institute of Sports Science, National Taiwan Sport University, Taoyuan, Taiwan; ^5^School of Post-baccalaureate Chinese Medicine, Tzu Chi University, Hualien, Taiwan; ^6^Center of Sports Medicine, Dalin Tzuchi Hospital, The Buddhist Tzuchi Medical Foundation, Chiayi, Taiwan; ^7^Emergency Department, Dalin Tzuchi Hospital, The Buddhist Tzuchi Medical Foundation, Chiayi, Taiwan; ^8^Division of Allergy, Immunology and Rheumatology, Dalin Tzuchi Hospital, The Buddhist Tzuchi Medical Foundation, Chiayi, Taiwan; ^9^School of Medicine, Tzu Chi University, Hualien, Taiwan; ^10^Department of Occupational and Environmental Medicine, National Cheng Kung University Hospital, Tainan, Taiwan; ^11^Occupational Safety, Health, and Medicine Research Center, National Cheng Kung University, Tainan, Taiwan; ^12^Department of Nursing, Tzu Chi University of Science and Technology, Hualien, Taiwan; ^13^Department of Medical Research, Dalin Tzuchi Hospital, The Buddhist Tzuchi Medical Foundation, Chiayi, Taiwan

**Keywords:** rheumatoid arthritis, hearing loss, Chinese herbal medicines, risk, cohort study

## Abstract

**Objectives:** Patients with rheumatoid arthritis (RA) are at a higher risk of extra-articular manifestations, especially hearing loss (HL). Although Chinese herbal medicines (CHM) are proven safe and effective treatments for inflammatory conditions, the effect of CHM use on HL in RA patients is unknown. This cohort study aims to determine the relationship between CHM use and the subsequent risk of HL among RA patients.

**Methods:** From health insurance claims data in Taiwan, a total of 6,905 persons aged 20–80 years with newly-diagnosed RA in 2000–2009 were identified. Of these, we recruited 2,765 CHM users and randomly selected 2,765 non-CHM users who matched with the users by the propensity score. Both cohorts were followed up until the end of 2012 to estimate the incidence of HL. Cox proportional hazards regression was used to estimate the adjusted hazard ratio (HR) for HL.

**Results:** The incidence of HL was lower in the CHM users than in the comparison cohort (8.06 vs. 10.54 per 1,000 person-years) (adjusted HR, 0.77; 95% CI, 0.63–0.94). Those who received CHM for more than 2 years had the greatest benefit against the onset of HL, with over 50% risk reduction. Prescriptions of Hai Piao Xiao, Yan Hu Suo, San-Qi, Huang Qin, Dang Shen, Jia-Wei-Xiao-Yao-San, Shu-Jing-Huo-Xue-Tang, and Dang-Gui-Nian-Tong-Tang were found to be associated with a reduced risk of HL.

**Conclusions:** Our findings suggest that adding CHM to conventional therapy may reduce the subsequent risk of HL in RA patients. Prospective randomized trials are recommended to further clarify whether the association revealed in this study supports such a causal relationship.

## Key Points

- Despite recent improvements in rheumatology treatment, those with rheumatoid arthritis (RA) are found to experience a higher risk of extra-articular manifestations, such as hearing loss (HL), which has been postulated to affect cognitive function.
- Recently, Chinese herbal medicines (CHM) are regarded as a popular adjunctive treatment for patients with RA; nevertheless, the association between CHM and risk of HL among them is still unknown.
- This study utilized nationally representative longitudinal data and revealed the therapeutic effect of CHM on the subsequent risk of HL among RA patients.
- The most prominent effect is further observed among those receiving CHM for more than 2 years, with an over 50% reduction in HL incidence.

## Introduction

Rheumatoid arthritis (RA) is a common autoimmune disease that attacks multiple joints throughout the body. The clinical hallmarks of RA include swelling, tenderness, and damage in synovial joints, that ultimately cause progressive functional limitations and physical disability in the affected patients. According to an analysis by the National Health Interview Survey 2011–2013, arthritis/rheumatism ranked among the top three conditions reported to cause work disability, regardless of age or sex, consequently imposing a tremendous socioeconomic burden (1). As estimated by a comprehensive nationwide database, the annual direct medical costs for one RA patient in the USA were approximately three times greater than those for a person without RA ($20,919 vs. $7,197) (2).

The chronic inflammation associated with RA can also affect other parts of the body, including the skin, eyes, and lungs. Recent studies have investigated extra-articular manifestations of RA, including hearing loss (HL) (3). One meta-analysis of 12 studies demonstrated that individuals with RA had more than double the likelihood of developing HL than did the general public (4). The elevated expression of pro-inflammatory mediators such as interleukin-6 (IL-6) and matrix metalloproteinase-3 was presumed to link these two disorders (5). HL can have social psychological consequences, as the resulting deficits in speech comprehension and difficulty communicating can cause the individual to withdraw from social activities (6). A recent longitudinal follow-up study using a national population sample showed that individuals with HL were over four times more likely to die than the general population (7). Thus, when managing patients with RA, preventing or treating HL is critical.

Over the past decade, Chinese herbal medicines (CHM) have become a commonly-used complementary therapy for patients with HL (8). Several herbs were proven to improve auditory function by normalizing the blood supply to the cochlea, decreasing the accumulation of toxic and inflammatory substances, and increasing the antioxidant defense in sensory hair cells (9), all of which play distinct roles in otoprotective mechanisms. For example, a compound from the root of Dan-Shen promotes hair cell survival and antioxidant protection by decreasing nuclear factor kappa beta (NF-κB) signaling (10–12). The results of these studies suggested that the integration of CHM into conventional treatments may be a useful measure to prevent the development of HL.

To date, the studies of the otoprotective effects of CHM have been conducted in relatively small patient cohorts, thus placing the broader applicability of the study results in question (8). Most importantly, no study has investigated the long-term effect of CHM use on HL prevention in RA patients. The range of potential medical resources available to the management of HL in RA patients may be limited by the paucity of available evidence. Therefore, using a nationwide medical claims database, this study aims to determine the effect of CHM use on the development of HL among RA patients.

## Methods

### Data Source

The data used in this retrospective cohort study were obtained from the Longitudinal Health Insurance Database (LHID), a sub-dataset of the National Health Insurance Research Database (NHIRD) of Taiwan made up of 1 million randomly-sampled people who registered with the National Health Insurance (NHI) program in 2000. Constructed using a multistage stratified systematic sampling method, LHID has also been demonstrated that patients registered in it have similar age and sex distributions to the whole Taiwanese population (13). This cohort database includes (i) personal information; (ii) health insurance claims data; (iii) diagnostic codes; (iv) prescription drug registry; (v) socioeconomic data; and (vi) medical examination information on persons under the NHI program. This study was conducted in accordance with the Helsinki Declaration and was approved by the local Institutional Review Board and Ethics Committee of Buddhist Dalin Tzu Chi Hospital (No. B10004021-2).

### Definition of Participants

**Figure 1:**
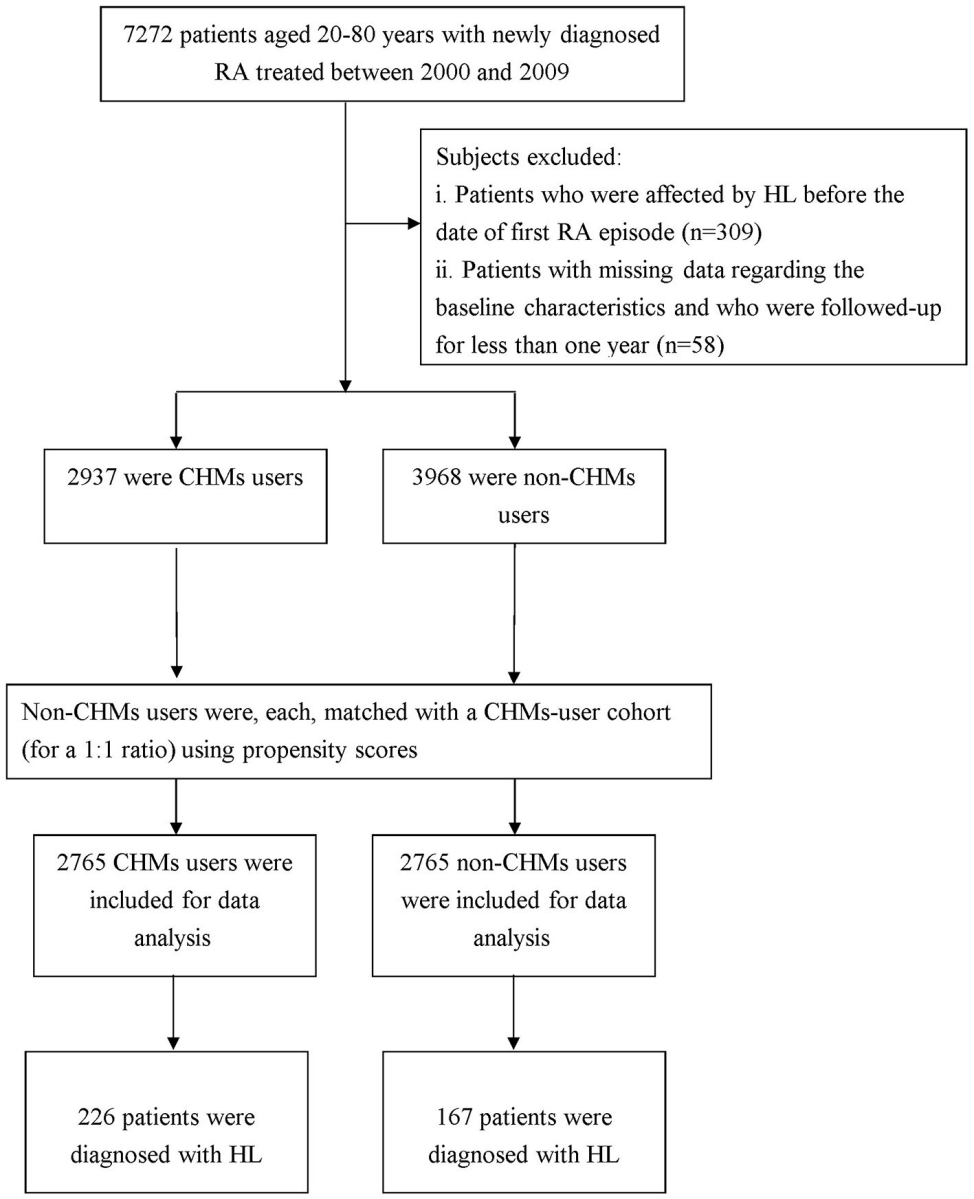
Flowchart showing the method of selecting and following study subjects.

To be included in this study, patients had to have a relevant code designated by the International Classification of Disease, Ninth Revision, Clinical Modification (ICD-9-CM). As shown in the Figure 1, we initially identified those who sought ambulatory health care services between 2000 and 2009 for newly diagnosed RA (ICD-9-CM code: 714.0). These patients were further linked to the catastrophic illness registry to ensure a valid diagnosis. The catastrophic illness certificate is granted based on formal diagnoses issued by physicians for conditions such as schizophrenia, mood disorders, autoimmune disorders, and cancer. The date when the patient gained approval for catastrophic illness registration was considered the index date. Only those aged 20–80 years were included. We then excluded those who had a previous diagnosis of HL before the first RA diagnosis (*n* = 309) and those who were not followed up for one complete year after RA diagnosis (*n* = 58), allowing temporal information to be reviewed. Patients were recognized as having HL if they had at least three ambulatory visits for treatment or if they had been hospitalized due to HL, as reflected in the use of ICD-9-CM codes 388.2, 388.4, 389.2, 389.9, 389.00, 389.10, or 389.12. The final cohort comprised 6,905 RA patients.

We used the frequency of visits to Chinese medicine physicians to determine whether the enrollee ever used CHM treatment. Those who used CHM to treat RA for more than 30 days were deemed CHM users (*n* = 2,937), and those treated for 30 days or less were considered non-CHM users. Because enrollees with a history of frequent CHM use would have a greater tendency to receive CHM than the general population, we matched each CHM user to one without CHM treatment by propensity scores to reduce potential biases. The propensity score was calculated using logistic regression based on patient demographics and baseline comorbidities at enrollment. The index date for the follow-up period was the date of CHM treatment initiation or, for non-CHM users, the date of the initial RA onset, corrected by immortal time bias (14). The follow-up time, in person-years, was calculated until the date of HL diagnosis or until censoring due to death, withdrawal from the insurance system, or loss to follow-up.

### Measured Covariates

The covariates considered in this survey included sociodemographic characteristics and comorbidities. Basic sociodemographic factors included age, sex, income (estimated using insurance premium), and urbanization level. Monthly income was subdivided into 3 levels, as follows: ≤ 17,880 New Taiwan Dollar (NTD); 17,881–43,900 NTD; and ≥ 43,901 NTD. Urbanization levels were divided into the following 3 strata: urban (levels 1–2), suburban (levels 3–4), and rural (levels 5–7), with a lower level indicating greater urbanization (15). Because previous illnesses may affect susceptibility to HL (8), the Charlson Comorbidity Index (CCI) score was used to summarize the influence of baseline comorbidities (16). To account for the use of conventional therapy, we defined patients who were prescribed with corticosteroids or disease-modifying anti-rheumatic drugs for 6 months or more as receiving conventional therapy. These data were obtained from both ambulatory care and inpatient hospitalization claims during the year preceding cohort entry. To avoid double counting and possible over-adjustment in the regression model, RA had been excluded from the CCI score estimation.

### Statistical Analysis

All analyses were performed using SAS Version 9.3 software (SAS Institute Inc, Cary, NC, USA) with a two-tailed significance level set at 0.05. We first compared the distributions of sociodemographic data and comorbidities between two groups using the chi-square test and Student's *t*-test. Cox proportional hazards regression analysis was then used to evaluate the risk of developing HL based on the exposure of CHM use, expressed as the hazard ratio (HR). An unadjusted model and a fully adjusted model (with all baseline covariates included) were developed. To further test the robustness of the relationship between CHM use and sequent HL risk, we divided the CHM users into three subgroups: CHM use duration, 31–365 days; CHM use duration, 366–730 days; and CHM use duration >730 days. The Kaplan–Meier method was used to estimate the cumulative risk of HL in each group, and the difference between groups was assessed using the log-rank test. The proportional hazards assumption was examined by plotting the log [–log (survival function)] vs. the log (survival time).

We also performed two sensitivity analyses using separate procedures. First, we included only the RA patients who reported no comorbidities. Second, we added the prescription of biological agents for 6 months or longer to be a surrogate for RA severity into the regression model.

## Results

Among the included study participants, following the propensity score matching as aforesaid, 2,765 participants were classified into the CHM users and 2,765 were thus selected into the non-CHM users. After matching, no significant differences were observed between the two groups with respect to age, sex, monthly income, urbanization level of the residential area, receiving conventional therapy or comorbidities (Table 1).

**Table 1:** Demographic data and selected comorbidities of study participants.

**Variables**	**Non-CHM users**	**CHM users**	***p***
	***n* = 2,765 (%)**	***n* = 2,765 (%)**	
**Age (yr)**			0.44
≤ 50	1,116 (40.4)	1,113 (40.3)	
>50	1,649 (59.6)	1,652 (59.7)	
Mean (SD)	54.08 ± 14.5	54.02 ± 13.5	
**Sex**			0.74
Female	2,035 (73.6)	2,046 (74.0)	
Male	730 (26.4)	719 (26.0)	
**Monthly income**			0.81
Low	1,204 (43.5)	1,186 (42.9)	
Medium	1,462 (52.9)	1,473 (53.3)	
High	99 (3.6)	106 (3.8)	
**Residential area**			0.75
Urban	1,574 (56.9)	1,551 (56.1)	
Suburban	425 (15.4)	423 (15.3)	
Rural	766 (27.7)	791 (28.6)	
**Conventional therapy**			0.13
Yes	2,027 (73.3)	2,076 (75.1)	
No	738 (26.7)	689 (24.9)	
**CCI**			0.43
Mean ± SD	6.03 ± 8.1	6.02 ± 8.5	

*CHM, Chinese herbal medicines; SD, standard deviation; CCI, Charlson-Deyo Comorbidity Index*.

**Figure 2:**
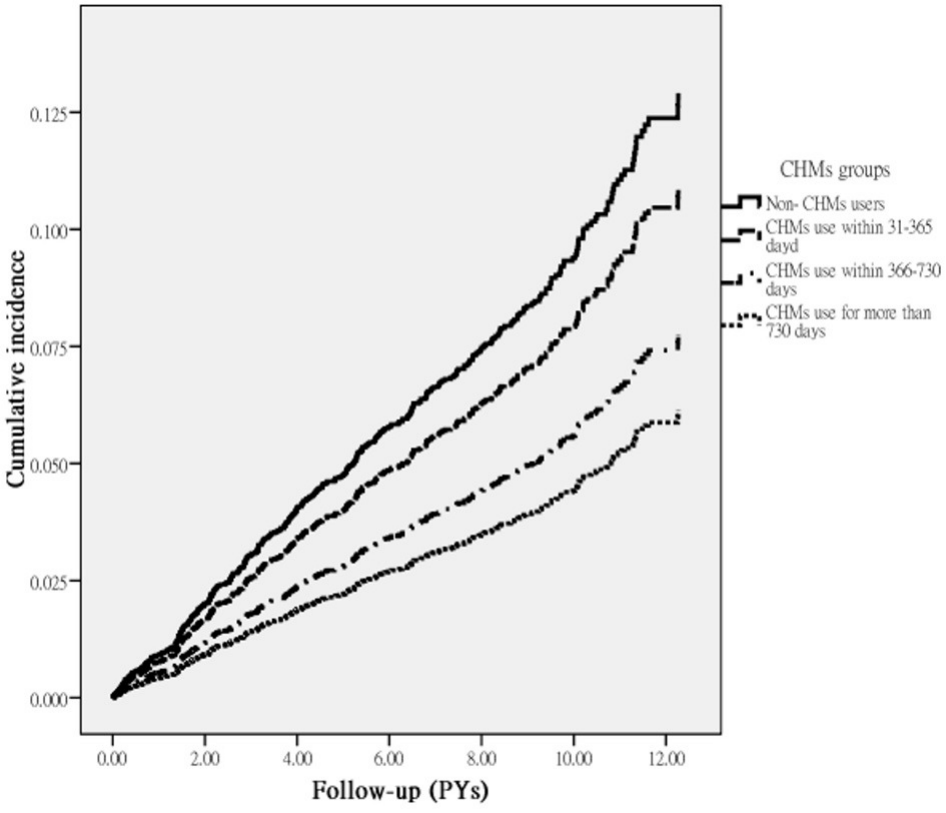
Cumulative incidence of HL in RA patients with and without receiving CHM treatment during the study period (Log-rank test, *p* < 0.001).

A total of 393 first episodes of HL occurred in the RA cohort over an observation period of 42,191.95 person-year (PY) in 5,530 patients. We observed that CHM users had a lower incidence of HL (8.06/1,000 PY) compared to the non-CHM users (10.52/1,000 PY), with an adjusted HR of 0.77 (95% CI: 0.63–0.92) (Table 2). In addition, those receiving CHM for more than 730 days were at an even lower risk of HL (54%) than those without CHM use. The Kaplan–Meier analysis of survival by days of CHM use revealed a statistically significant difference in the survival rate free from HL across the three groups of CHM users (*p* < 0.001) (Figure 2).

**Table 2:** Risk of HL for RA patients with and without CHM use.

**Patient group**	**Event**	**PY**	**Incidence (/1000 PY)**	**Crude HR (95% CI)**	Adjusted HR^*^ (95% CI)
Non-CHM users	226	21,472.89	10.52	1	1
**CHM users**	167	20,719.06	8.06	0.76 (0.64–0.94)	0.77 (0.63–0.92)
CHM use within 31–365 days	144	16,593.81	8.68	0.82 (0.67–0.98)	0.83 (0.67–0.99)
CHM use within 366–730 days	15	2,459.31	6.10	0.57 (0.34–0.96)	0.57 (0.34–0.96)
CHM use >730 days	8	1,665.93	4.80	0.45 (0.23–0.93)	0.46 (0.23–0.92)

* *Model adjusted for age, sex, urbanization level, monthly income, conventional therapy, and Charlson-Deyo Comorbidity Index*.

Collectively, a more beneficial effect of CHM on HL was observed among younger patients (Table 3). Multivariable stratified analysis showed that the benefit of CHM therapy in reducing the incidence of HL was more predominant among females, with an adjusted HR of 0.68 (95% CI, 0.55–0.86) (Table 3). The 10 most commonly prescribed herbal formulas for RA treatment are shown in Table 4. Of these, Hai-Piao-Xiao, Yan Hu-Suo, San-Qi, Huang-Qin, Dang-Shen, Jia-Wei-Xiao-Yao-San, Shu-Jing-Huo-Xue-Tang, and Dang-Gui-Nian-Tong-Tang were found to be associated with a significant reduction in the HL risk (Table 4).

**Table 3:** Incidence and risk of HL among RA patients with and without CHM use by sex and age.

**Variables**	**Non-CHM users**			**CHM users**			**Crude HR (95% CI)**	**Adjusted HR (95% CI)**
	**Case**	**PY**	**Incidence^†^**	**Case**	**PY**	**Incidence**		
**Sex**								
Female	179	15,449.44	11.59	119	15,149.27	7.86	0.68 (0.54–0.85)	0.68^¥^ (0.55–0.86)
Male	47	6,023.45	7.80	48	5,569.79	8.62	1.07 (0.75–1.49)	1.06^¥^ (0.71–1.52)
**Age (year)**								
≤ 50	70	8,844.38	7.91	49	8,622.35	5.68	0.72 (0.49–0.99)	0.69^*^ (0.48–0.98)
>50	156	12,628.51	12.35	118	12,096.71	9.75	0.80 (0.63–1.01)	0.79^*^ (0.63–1.01)

¥ *Model adjusted for age, urbanization level, monthly income, conventional therapy, and Charlson-Deyo Comorbidity Index*.

† *Per 1000 PY*.

* *Model adjusted for sex, urbanization level, monthly income, conventional therapy, and Charlson-Deyo Comorbidity Index*.

**Table 4:** Risk of HL associated with use of the 10 most-used single-herb and multi-herb CHM products for RA patients.

**Chinese herbal medicine**	**Number of prescriptions**	**Crude HR (95% CI)**	Adjusted HR^*^ (95% CI)
**Single-herb products**			
Yan-Hu-Suo	6,527	0.72 (0.53–0.98)	0.71 (0.52–0.96)
Dan-Shen	4,828	0.69 (0.50–0.95)	0.67 (0.48–0.92)
Ji-Xue-Teng	4,596	0.89 (0.65–1.20)	0.81 (0.59–1.12)
Huang-Qin	4,235	0.67 (0.50–0.93)	0.62 (0.45–0.86)
Bei-Mu	4,181	0.95 (0.71–1.34)	0.94 (0.67–1.29)
Da-Huang	3,758	0.76 (0.53–1.09)	0.72 (0.51–1.03)
Hai-Piao-Xiao	2,783	0.56 (0.40–0.79)	0.53 (0.37–0.74)
Mu-Xiang	2,115	0.95 (0.65–1.40)	0.92 (0.63–1.37)
San-Qi	2,082	0.63 (0.44–0.90)	0.60 (0.42–0.86)
Chuan-Niu-Xi	1,650	0.78 (0.52–1.19)	0.76 (0.50–1.15)
**Multi-herb products**			
Shu-Jing-Huo-Xue-Tang	7,303	0.79 (0.58–1.01)	0.77 (0.59–1.04)
Jia-Wei-Xiao-Yao-San	6,968	0.74 (0.54–0.98)	0.63 (0.46–0.86)
Du-Huo-Ji-Sheng-Tang	4,962	0.88 (0.63–1.23)	0.88 (0.62–1.20)
Shao-Yao-Gan-Cao-Tang	4,625	0.71 (0.53–0.97)	0.67 (0.49–0.92)
Ge-Gen-Tang	4,596	0.89 (0.65–1.19)	0.88 (0.59–1.11)
Dang-Gui-Nian-Tong-Tang	3,972	0.75 (0.54–0.99)	0.71 (0.51–0.98)
Chuan-Xiong-Cha-Tiao-San	3,939	0.89 (0.64–1.24)	0.83 (0.60–1.15)
Xue-Fu-Zhu-Yu-Tang	3,583	0.95 (0.68–1.34)	0.93 (0.66–1.30)
Ping-Wei-San	3,323	0.84 (0.60–1.19)	0.83 (0.59–1.17)
Gan-Lu-Yin	3,188	0.73 (0.52–1.01)	0.72 (0.52–1.00)

* *Model adjusted for age, sex, urbanization level, monthly income, conventional therapy, and Charlson-Deyo Comorbidity Index*.

In the first sensitivity analysis, when only the RA patients who reported no comorbidities were included in the analysis, we found that CHM use was still associated with a lower incidence rate of HL, with an adjusted HR of 0.81 (95% CI, 0.75–0.97). In the second sensitivity analysis, 52.3% (1,446/2,765) of the CHM users and 52.7% (1,458/2,765) of the non-CHM users had received biological agents for 6 months or longer. When this factor was added to the model, we found CHM use was still associated with a lower incidence rate of HL, with an adjusted HR of 0.79 (95% CI, 0.68–0.96).

## Discussion

Hearing impairment in RA patients can decrease social interactions and compliance with healthcare treatment, thus worsening the manifestations of RA. Because HL is more common among RA patients than in the general population, more effective treatments are needed to prevent HL. In this evidence-based cohort study, we observed that those receiving CHM treatment had a 23% lower risk of HL than did those not using CHM. In those receiving CHM for more than 2 years, the risk of HL decreased by over 50%. The dose–response relationship observed between the intensity of CHM use and the decrease in the risk of HL supports a causal association between CHM use and HL prevention. No previous study has investigated the long-term impact of CHM on the HL risk in RA patients. The positive correlation between CHM use and decreased risk of HL observed herein is consistent with previous reports and bolsters a growing body of evidence on this topic (8, 9). The proposed mechanisms by which the prescribed herbal products protect against the onset of HL include reduction in blood viscosity, regulation of the inflammatory response, improvement of lymph circulation, and enhancement of the excitability and conductivity of the auditory nerve (5, 8), all of which exert a beneficial effect on cochlear microcirculation.

We found that younger patients benefited more from CHM use in lowering the HL risk. Compared to older patients, younger patients often have a more positive attitude toward their medical condition as well as more and better psychosocial and coping resources to rely upon (17), all of which might enhance the protective effect of CHM on the auditory system. We found that female patients also benefited more from CHM use. Compared to males, females often display better health literacy, more willingness to adhere to strict medical procedures, and more positive attitudes about self-care (18). In addition, besides suppressing the inflammatory cytokines that play decisive roles in the generation of neurodegenerative disorders (19), the female hormone estrogen was further found to protect the inner ear by amplifying the expression of insulin-like growth factor 1 in the cochlea, which have been shown to be responsible for cell proliferation, metabolism, and anti-apoptotic cellular responses (20).

Another focal aim of this study was to identify the specific CHM constituents that were likely related to the reduced risk of HL in RA patients. Of the commonly used single-product CHM to treat RA, a total of five herbs were found to be associated with a lower subsequent risk of HL. Studies in animal models indicate that extracts of Yan-Hu-Suo and Dan-Shen modulate the induction of IL-6 or tumor necrosis factor-α by abating NF-κB signaling (21, 22). Recent evidence implicates these inflammatory markers in the pathogenesis of central auditory disorders (8, 23).

We observed that the use of Huang-Qin was associated with a decreased risk of HL. Baicalin and baicalein, major flavonoids present in this formula, have attracted considerable attention because of their anti-inflammatory activity and antioxidant effects (9). Zhang et al. reported that baicalin use ameliorated the degeneration of brain and auditory nerve in rats with noise-induced HL by targeting the toll-like receptor 4/NF-κB signaling pathway (24). Activation of this signaling pathway may induce sensory cell degeneration and cochlear dysfunction (25), thus promoting the onset of HL.

Our study results indicate that the use of Hai-Piao-Xiao might lessen the subsequent susceptibility to HL among RA patients. A previous *in vitro* study showed that Hai-Piao-Xiao extract profoundly normalized the blood supply to the cochlea and increased the antioxidant defense in sensory hair cells (26), consequently modifying susceptibility to hearing disorders. A strong association between San-Qi and reduced HL risk was also observed in the present study. This herb is a species of the genus Panax and is commonly used to stimulate blood circulation to promote physiological function in the nervous or immune system. San-Qi activates the AKT/Nrf2-mediated redox pathway (27, 28), thus resulting in more efficient neurotransmitter function.

Of the commonly prescribed multi-herb products, Jia-Wei-Xiao-Yao-San may lower the risk of HL. In a rodent model, this formula was observed to increase synaptic plasticity and decrease the levels of inflammatory markers by activating intracellular signaling pathways, especially the brain-derived neurotrophic factor (29). This neurotrophic factor has been found in many areas of the brain and plays an important role in supporting the formation of memories and auditory processing (30). These phenomena may underlie the positive effect of Jia-Wei-Xiao-Yao-San observed in this study.

Consistent with the findings of earlier research (31), this study also revealed that Dang-Gui-Nian-Tong-Tang was the most frequently used Chinese herbal formula for treating rheumatic disorders. The positive association between Dang-Gui-Nian-Tong-Tang use and a lower incidence of HL may result from its reported effects of increasing blood circulation and inhibiting inflammatory cytokines (32), processes that have been implicated in the development of hearing disorders. Regarding Shao-Yao-Gan-Cao-Tang, we speculate that this natural product may mitigate the onset of HL through its proven abilities of inhibit the development of inflammatory conditions and slow the degeneration of neurons via a variety of pharmacological effects, including antioxidant, anti-inflammatory, and anti-apoptotic activities (33).

While our study is the first to investigate the relationship between CHM use and subsequent risk of HL among RA patients, some limitations should be considered. First, although a positive association was observed between CHM use and a lower risk of HL, potential misclassification may have influenced the results because of the use of ICD-9-CM diagnostic codes alone. To minimize this, we enrolled only patients with new-onset RA or HL, and only after the patients had at least 3 outpatient visits with consistent diagnoses or at least one inpatient admission due to RA or HL. It should also be noted that the NHI of Taiwan randomly reviews the charts and audits medical charges to verify the accuracy of claims (13). Additionally, the coding approach and data availability were similar between the two groups, and any misclassification, if exists, was likely to have been non-differential, thus resulting in an underestimate (dilution) of the true strength of an association between exposure and disease. Second, a reliable index of RA severity was unavailable from the LHID, and failure to control for this factor may bias the findings. To address this potential problem, we performed two sensitivity analyses: one limited the analysis to the patients without comorbidity, and the other added the prescription of biological agents for 6 months or longer, a common surrogate used for RA severity (34, 35), to the analyses. These sensitivity analyses support that disease severity was not likely to introduce a remarkable effect on our conclusion—adding CHM to conventional therapy may reduce the subsequent risk of HL in RA patients. Third, the LHID lacks information on certain potential confounders such as social network relationships, smoking, alcohol intake, personality attributes, laboratory data, and education level. Research addressing these variables is needed to shed further light on these preliminary findings. Fourth, although our study revealed a substantial beneficial effect of CHM use on reducing HL among RA patients, it must be recognized that participants were not initially randomly categorized into users and non-users and were only recruited from a single country. Therefore, caution should be taken when interpreting the findings. Randomized controlled trials of cohorts including patients from additional countries are recommended to corroborate the present findings and to clarify the mechanisms underlying the clinical benefits of CHMs. Fifth, the NHI program in Taiwan only pays for Chinese herbal products prescribed by Chinese medicine physicians, and not for over-the-counter medicine (36). These limitations notwithstanding, this study also possessed several strengths, including the completeness of the records of hospital diagnoses and prescription claims, the minimal risk of selection bias and loss to follow-up, and the large population of both men and women. These factors provide sufficient power to conduct detailed analyses, which is unique given the relatively low incidence of RA in the general population. As to the safety of CHM, following a careful review of the Taiwan National Adverse Drug Reactions Reporting System (37), we found that there were no severe drug interaction effects occurring among Taiwanese RA patients who used the concomitant treatments of Western medicines and Chinese herbal products so far.

## Conclusion

This study demonstrated that RA patients who used CHM may have a 23% lower risk of developing HL than did those without CHM use. Although a beneficial effect of CHM use on the risk of HL was found in this survey, future prospective randomized trials that overcome the limitations of this study are needed to provide more definite evidence of the association suggested herein. If the effect can be confirmed, efforts should be made to identify other groups of patients who may also benefit from it. Further studies exploring additional benefits of CHM on rheumatoid joint symptoms are also warranted.

## Data Availability Statement

The raw data supporting the conclusions of this article will be made available by the authors, without undue reservation.

## Ethics Statement

The studies involving human participants were reviewed and approved by Institutional Review Board and Ethics Committee of Buddhist Dalin Tzu Chi Hospital. Written informed consent for participation was not required for this study in accordance with the national legislation and the institutional requirements.

## Author Contributions

H-HL, HL, and T-YT: study concept and design. M-CL and T-YT: acquisition of data. HL, T-YT, and H-RG: data analysis. M-CL, T-YT, and W-LF: project management. W-JC, H-HL, HL, H-RG, T-YT, M-CL, and W-LF: writing. All authors contributed to the article and approved the submitted version.

## Conflict of Interest

The authors declare that the research was conducted in the absence of any commercial or financial relationships that could be construed as a potential conflict of interest.
